# Breaking bad news to cancer patients in times of COVID-19

**DOI:** 10.1007/s00520-021-06167-z

**Published:** 2021-03-29

**Authors:** Helen Hauk, Jürg Bernhard, Meghan McConnell, Benny Wohlfarth

**Affiliations:** 1 Department of Visceral Surgery and Medicine, Inselspital, Bern University Hospital, University of Bern, Bern, Switzerland; 2 Department of Medical Oncology, Inselspital, Bern University Hospital, University of Bern, Bern, Switzerland; 3 Department of Innovation in Medical Education, Department of Anesthesiology and Pain Medicine, University of Ottawa, Ottawa, Ontario Canada

**Keywords:** Breaking bad news, COVID-19, Social distancing

## Abstract

Breaking bad news is a mandatory provision in the professional life of nearly every physician. One of its most frequent occasions is the diagnosis of malignancy. Responding to the recipients’ emotions is a critical issue in the delivery of unsettling information, and has an impact on the patient’s trust in the treating physician, adjustment to illness and ultimately treatment. Since the World Health Organization (WHO) declared COVID-19 a pandemic on March 11, 2020, several measures of social distancing and isolation have been introduced to our clinical setting. In the wake of these restrictions, it is important to reexamine existing communication guidelines to determine their applicability to face-to-face counseling in the context of social distancing, as well as to new communication technologies, such as telemedicine. We address these issues and discuss strategies to convey bad news the most empathetic and comprehensible way possible.

Dear Editor,

Breaking bad news is a necessary competency for nearly every physician. Life-altering events, like the diagnosis of malignancy, can be associated with various emotions like shock, fright, sadness, or reactions of avoidance, denial, or dissociation [1, 2]. Responding to such emotions is important when delivering unsettling information and has an impact on the patient’s trust in the physician, adjustment to illness, and treatment [3–6].

Under the current COVID-19 pandemic, several measures of social distancing and isolation have been introduced to our clinical setting and make it mandatory to reevaluate current communication in face-to-face counseling under the burden of social distancing, as well as towards new communication technologies, such as telemedicine.

## Restrictions of visitors

The involvement of family members or friends plays a critical role for many patients and within established guidelines for breaking bad news. Such persons not only provide emotional support but also help patients capture critical information [7–10]. Furthermore, the aftermath of miscommunication has been associated with various detrimental consequences, such as decreased adherence to clinician recommendations, greater psychological distress, and increased social withdrawal [3, 4, 11]. Restrictions of supportive persons may augment patients’ cognitive, behavioral, or emotional difficulties while receiving bad news.

To counteract, we emphasize the importance of alternative strategies to involve supportive persons in any clinically relevant discussions. Telecommunication devices (e.g., phone or video conferencing) allow patients’ support personnel to participate in these conversations despite physical absence. Additionally, alternative meetings in the immediate surroundings of the hospital can provide a valuable extension for the overall support of the patient.

Extending the support-network by involving a nurse, social worker, chaplain, or psychologist in the communication may here be even more important than it was in non-pandemic times. Furthermore, given that some patients perceive greater empathy in the process of a bad news disclosure from their general practitioner [12], involving the patient’s family doctor may improve difficult conversations in hospital settings.

Generally, it is important to evaluate the patient’s understanding of what is being discussed. Audio or video recordings, which have been shown to improve patients’ ability to recall information during physician-patient consultations [13], represent important supportive tools for the patient. Lastly, it is essential to assess the patient’s understanding of the current restrictions in advance and adapt the setting accordingly.

## Restrictions of physical contact

Most patients prefer that bad news be given in person and without physical barriers [14–16]. Positioned close to the patient, the bearer of bad news can more easily respond to emotional cues, for example, by touching the patient’s arm or hand if appropriate [17]. Indeed, research has shown that physical contact during such situations can have a positive effect on the patient [15, 18]. Restrictions of physical contact and distance make it more difficult for healthcare providers to respond to patients’ emotions in an empathetic, compassionate way.

However, when explicitly pointed out by the physician as a precautionary measure due to the pandemic, such actions are less likely to be perceived as unfriendly and patients may recognize such words as a sign of solidarity. Such restrictions also hold the opportunity for doctors and patients to relate to one another under the pandemic. This can have a beneficial impact on the relationship and patient outcomes, as confiding communication has been associated with better patient care and adjustment to illness and treatment [3–6]. Therefore, we propose that physicians start conversations by making a short encouraging statement regarding current restrictions in the local clinical setting. Additionally, by asking how he or she is dealing with the pandemic situation, the physician may learn about the patient’s personal circumstances before opening the main dialogue. This may give an indication of the patient’s preferred level of comprehensiveness of information and ability to cope with the upcoming news, two factors that may vary substantially [5, 19, 20]. Besides all these interpersonal aspects, a friendly, non-technical ambience (e.g., pictures, plants) may help to distract from the restriction measures.

## Restrictions of countenance

Facial masks, worn by both patients and healthcare providers [21], preclude a significant portion of facial expressions. Researchers have argued that 93% of communication consists of nonverbal cues such as facial expressions, gestures, and body movement [22]. Nonverbal communication is particularly important in conveying emotions and has a decisive impact on how individuals perceive and interpret the message [23]. Facial expressions, as well as gestures like posture, nodding of the head, and movement of the extremities, provide patients with information on physicians’ interest and empathy [24]. Restrictions of countenance can therefore make it more difficult for the physician to convey warmth, reassurance, and compassion. Given the importance of compassion in patient-centered communication [25, 26], restrictions may affect not only the doctor-patient relationship but also the adherence and therapeutic outcome. According to our observation, current restrictions of facial expression led to a shift of increased gesticulation, especially hand movements, by the bearer of bad news. Furthermore, complementing a more structured communication-style with open-ended questions may be reinforced by an inviting gesture to the patient. A conscious use of such gestures can reflect empathetic intention, support the lead of the conversation (i.e., hand the word over to the patient and take it back), and help the patient interpret the content of the verbal message. In turn, patients may feel encouraged to use gestures themselves.

Mutually, covered facial expressions of the patient make it difficult for the physician to respond adequately to the patient’s emotions and make patient-centered communication more challenging. Therefore, it is important to verbally address patients’ emotions, for example, to promote disclosure of their concerns or goals for care [27].

## Conclusion

Under the COVID-19 pandemic, the burden of breaking bad news to cancer patients has become even more challenging than it was in non-pandemic times. Physicians should emphasize thorough preparation of the consultation setting, minimize disruptive factors, and consider the aforementioned caveats and strategies as mentioned above and in the corresponding table (Table 1).

**Table 1:** Guidance to optimize breaking bad news to patients under isolation measures

1. Minimize disruptive factors in the clinical setting that could hamper the delivery of bad news (e.g., turn off cellphones, sign-out beepers).	
2. Use shared affection regarding the isolation measures to strengthen mutual trust and to learn about the patient’s personal circumstances.	
3. Deliberately focus on verbal skills to reinforce interest in the patient’s needs (e.g., via verbal connection statements).	
4. Emphasize exploratory questions to elaborate on the patient’s emotions and to verify his or her understanding of the conveyed information. Optionally: meet the patient through video conferencing to ensure non-verbal communication via facial expressions.	
5. Use alternative non-verbal signals, such as hand movements, to reflect your empathic intent, support the lead of the conversation and help the patient in understanding the actual content of the verbal message.	
6. Take advantage of a nurse, social worker, religious personnel, psychologist, or audio recording for the patient in the absence of close relatives or friends.	
7. Foster remote integration of patient’s relatives, friends or other key people (e.g., his/her general practitioner) by phone and video conferencing or alternative meetings in the immediate surroundings of the hospital.	
8. Create a friendly, non-technical ambience (e.g., pictures, plants) which may help to distract from the restriction measures.	

While originating from a hospital setting and in the light of the current pandemic, our article holds implications adaptable to many in- or outpatient clinical scenarios containing isolation measures.

## Data Availability

N/A
